# PrimeNet: rational design of Prime editing pegRNAs by deep learning

**DOI:** 10.1093/bib/bbaf293

**Published:** 2025-06-19

**Authors:** Xichen Liao, Qi Liu, Guohui Chuai

**Affiliations:** Department of Hematology, Tongji Hospital, Frontier Science Center for Stem Cell Research, Bioinformatics Department, School of Life Sciences and Technology, Tongji University, No. 1239 Siping Road, Yangpu District, Shanghai 200092, China; Shanghai Key Laboratory of Anesthesiology and Brain Functional Modulation, Clinical Research Center for Anesthesiology and Perioperative Medicine, Translational Research Institute of Brain and Brain-Like Intelligence, Shanghai Fourth People's Hospital, Frontier Science Center for Stem Cell Research, Bioinformatics Department, School of Life Sciences and Technology, Tongji University, No. 1239 Siping Road, Yangpu District, Shanghai 200092, China; National Key Laboratory of Autonomous Intelligent Unmanned Systems, Frontiers Science Center for Intelligent Autonomous Systems, Ministry of Education, No. 366 Shangke Road, Pudong New Area, Shanghai 201804, China; Department of Hematology, Tongji Hospital, Frontier Science Center for Stem Cell Research, Bioinformatics Department, School of Life Sciences and Technology, Tongji University, No. 1239 Siping Road, Yangpu District, Shanghai 200092, China; Shanghai Key Laboratory of Anesthesiology and Brain Functional Modulation, Clinical Research Center for Anesthesiology and Perioperative Medicine, Translational Research Institute of Brain and Brain-Like Intelligence, Shanghai Fourth People's Hospital, Frontier Science Center for Stem Cell Research, Bioinformatics Department, School of Life Sciences and Technology, Tongji University, No. 1239 Siping Road, Yangpu District, Shanghai 200092, China; National Key Laboratory of Autonomous Intelligent Unmanned Systems, Frontiers Science Center for Intelligent Autonomous Systems, Ministry of Education, No. 366 Shangke Road, Pudong New Area, Shanghai 201804, China; Department of Hematology, Tongji Hospital, Frontier Science Center for Stem Cell Research, Bioinformatics Department, School of Life Sciences and Technology, Tongji University, No. 1239 Siping Road, Yangpu District, Shanghai 200092, China; Shanghai Key Laboratory of Anesthesiology and Brain Functional Modulation, Clinical Research Center for Anesthesiology and Perioperative Medicine, Translational Research Institute of Brain and Brain-Like Intelligence, Shanghai Fourth People's Hospital, Frontier Science Center for Stem Cell Research, Bioinformatics Department, School of Life Sciences and Technology, Tongji University, No. 1239 Siping Road, Yangpu District, Shanghai 200092, China; National Key Laboratory of Autonomous Intelligent Unmanned Systems, Frontiers Science Center for Intelligent Autonomous Systems, Ministry of Education, No. 366 Shangke Road, Pudong New Area, Shanghai 201804, China

**Keywords:** CRISPR, gene editing, Prime editing, epigenetics, machine learning, deep learning

## Abstract

The rapid development of gene editing technology has revolutionized life science research and biotechnology applications. Prime editing, a precise gene editing tool, has shown promise in various applications, including disease research and therapeutic interventions. However, its suboptimal editing efficiency for extensive fragments and lack of predictive models have hindered its widespread adoption. Existing models exhibit low prediction accuracy and limitations, such as neglecting epigenetic factors that impact gene editing effects. To address these challenges, we developed PrimeNet, a novel prediction model that integrates significant epigenetic factors, including chromatin accessibility and DNA methylation. By incorporating data from multiple cell lines and introducing multiscale convolution and attention mechanisms, PrimeNet enhances the accuracy of predictions and generalization performance. Our results show that PrimeNet achieves a Spearman correlation coefficient of 0.94 and 0.82 on two datasets originated from HEK293T and K562 cell lines, respectively, outperforming existing models. This novel model has the potential to guide experimental design, enhance the success rate of gene editing, and reduce unnecessary experimental costs, thereby advancing the application of gene editing technology in genetic disease treatment and related fields.

## Introduction

The rapid development of gene editing technology has had a profound impact on life science research and biotechnology applications. Among the various gene editing tools that have emerged, Prime editing presents a remarkable degree of precision, capable of introducing programmed reverse transcriptase in conjunction with targeted RNA-directed nuclease, resulting in the ability to generate various distinct point mutations, insertions, and deletions, as well as combinations thereof [1]. This distinctive editing capacity has demonstrated considerable promise for diverse applications [2], including research on diseases and therapeutic interventions [3], the modification of model organisms [4], the functional screening of genetic variants [5], and agricultural production [6]. Prime editing has been demonstrated to circumvent the salient limitations of conventional CRISPR gene editing by circumventing double-strand breaks, mitigating the risk of off-targeting, facilitating diverse editing modalities, and enabling non-dividing cell applications. Nevertheless, Prime editing remains confronted with technical challenges, particularly its suboptimal editing efficiency for extensive fragments, given its more intricate process in comparison to prevalent CRISPR gene editing.

In order to enhance the efficacy of Prime editing, it is imperative to develop advanced predictive models. These models can assist researchers in reducing the number and cost of experiments, thereby maximizing the editing success rate. However, the existing models demonstrate a general lack of prediction accuracy. Early approaches, such as Easy-Prime, employ XGBoost-based gradient boosting trees trained exclusively on sequence features, achieving a low Spearman correlation of 0.67 across diverse datasets [7]. A similar observation is made with DeepPE, which introduces a single convolutional layer, comprising ten 3-nt filters applied separately to the target and PBS + RTT sequences, followed by a 1000-unit fully connected layer to predict efficiencies. Five-fold cross-validation yields Spearman correlations ranging from 0.47 to 0.81, albeit with substantial context-dependent variability [8]. Newly emerged tools including PRIDICT [9] and DeepPrime [10] have demonstrated enhanced prediction accuracy compared to previous models. PRIDICT utilizes an attention-based bidirectional recurrent neural network to effectively capture long-range dependencies within the pegRNA–target pair, achieving Pearson and Spearman correlations of 0.86 and 0.85. DeepPrime integrates convolutional layers with a bidirectional GRU to encode sequence context, alongside a separate four-layer perceptron module that extracts physicochemical bio-features, yielding median Pearson and Spearman correlations of 0.81 and 0.83. Notwithstanding the marked improvements in accuracy exhibited by PRIDICT and DeepPrime in comparison to earlier tools, critical limitations remain in place. The capacity of DeepPrime to predict edits of ≤3 bp is limited, thus constraining its applicability to larger insertions and deletions. Furthermore, both models fail to incorporate epigenetic information, such as chromatin accessibility, despite the existence of compelling evidence that these factors significantly influence Cas9–pegRNA binding and editing efficiency [11–15]. The absence of epigenetic context results in the systematic overestimation of efficiency in heterochromatic regions and the reduction of model generalizability across different cell types. Although PRIDICT2.0 has been demonstrated to be capable of applying fine-tuning to HEK293T and K562 cell lines, its scope remains restricted to these backgrounds [16].

Here, we developed a novel prediction model, PrimeNet, to address the limitations of existing models with respect to epigenetic factors. This model integrates significant epigenetic factors, including chromatin accessibility and DNA methylation, which directly affect the accessibility of target loci and the feasibility of editing. The integration of these features enables the model to more comprehensively grasp the intricate mechanisms underlying gene editing across diverse cell lines, thereby enhancing the accuracy of predictions. Additionally, by incorporating data from multiple cell lines, the model can acquire more extensive patterns and relationships, leading to an improvement in the accuracy of predictions for unseen cell lines. By introducing multiscale convolution and attention mechanisms, the model’s ability to capture different sequence features, as well as its generalization performance under various conditions, is significantly enhanced. In terms of performance, PrimeNet is among the most effective models of its kind. Through testing, it was found that PrimeNet can achieve a Spearman correlation coefficient of 0.94, indicating a very strong positive correlation between its prediction results and actual editing efficiency. This suggests that the utilization of PrimeNet can more effectively guide experimental design, enhance the success rate of gene editing, and reduce unnecessary experimental costs.

In summary, PrimeNet represents a notable step forward in the field of novel gene editing technology optimization. Its efficiency, accuracy, and flexibility position it as a valuable instrument for researchers and clinical practitioners, thereby fostering the advancement and application of gene editing technology in genetic disease treatment and other related fields.

## Materials and methods

### Data collection and processing

To address the limitations of existing models in adequately incorporating epigenetic factors, this study seeks to integrate these elements into the model construction process. Although the dataset used in DeepPrime [10] is relatively large in scale, it only includes editing events with a length of up to three bases, thereby constraining its applicability to broader editing tasks. Consequently, we utilized the PRIDICT dataset established by Mathis *et al*., which encompasses a more diverse range of editing types, with 107 165 samples from HEK293T cell lines and 18 742 samples from K562 cell lines. Detailed information in terms of the distribution of validly edited, unedited, and erroneously edited samples is provided in supplementary material (Supplementary Fig. S1). We then incorporated chromatin accessibility and DNA methylation data into the model to enable a more comprehensive and systematic modeling approach.

#### Integration of epigenetic information

To obtain information on DNA methylation and chromatin accessibility, reduced representation bisulfite sequencing (RRBS) and DNase data for this cell line were downloaded from the ENCODE database [17]. For datasets provided by different laboratories, the concatenation strategy was employed. That is, if either dataset shows methylation or chromatin accessibility at a site, the site is regarded as having the possibility of methylation or chromatin accessibility.

First, the unedited DNA sequence information was compared to the reference genome by Bowtie-2 [18], and the corresponding genomic coordinates were obtained. Subsequently, the collated RRBS and DNase data were correlated with these coordinates. For methylation features, a site is labeled as “Y” if it is methylated and “N” if it is unmethylated. For chromatin accessibility features, a site is labeled “Y” if it is chromatin accessible and “N” otherwise. This epistatic information was integrated into the model as the fourth and fifth channels of the pseudo-image, respectively. Furthermore, the locations of key functional domains in the sequence are already provided in the original data of Mathis *et al*., and no additional processing is required.

#### PegRNA encoding schema

**Figure 1:**
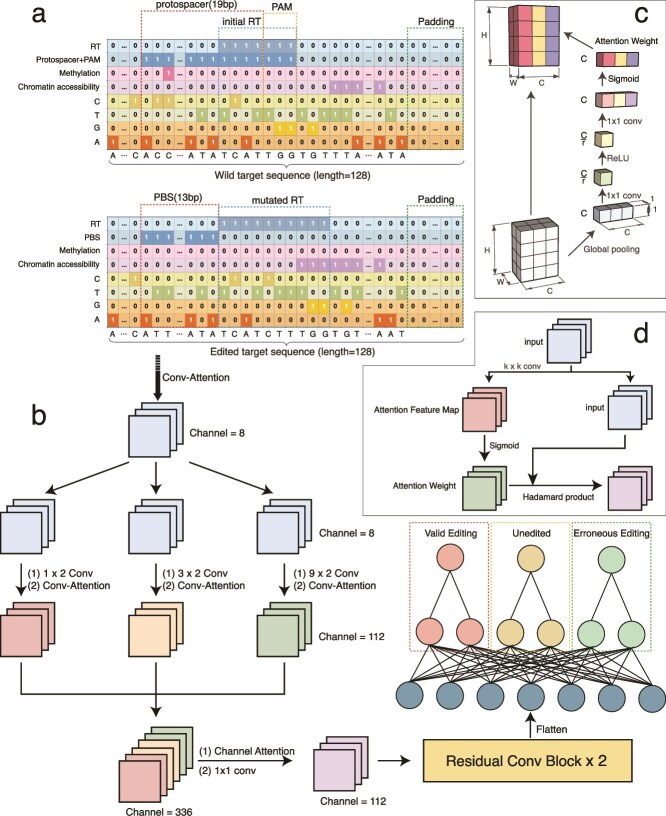
Implementation details of PrimeNet. **a** Schematic representation of data encoding. The figure illustrates two sequences, the wild target sequence and the edited target sequence, with their respective channels expanded to clearly indicate the meaning of each channel. **b** Model architecture diagram, highlighting changes in the number of channels to illustrate the functional principle of the multiscale convolution module. **c** Channel attention visualization; **d** Conv-attention visualization.

To integrate sequence information, epigenetic information, and functional region features into the inputs of a deep learning model, a multichannel pseudo-image-based encoding method was designed (Fig. 1a). This method utilizes multiple channels to encode DNA sequence, epigenetic information (e.g. DNase sensitivity and DNA methylation status), and important functional regions (including protospacer, PBS, and RT regions). Ultimately, this method generates a pseudo-image suitable for convolutional neural network processing. The pseudo-image comprises eight channels, with the specific meanings of each channel and the encoding method outlined below:

1. DNA sequence information (channels 0–3).

The bases of a deoxyribonucleic acid (DNA) sequence are encoded into four discrete channels, each corresponding to the position of a specific base. For instance, if the current base is A, channel 0 represents position 1, and the remaining channels are assigned to 0. Similarly, G, T, and C are mapped to channels 1, 2, and 3, respectively.

2. Epigenetic information (channels 4 and 5).

Epigenetic information including DNase sensitivity and DNA methylation status:

- DNase sensitivity (channel 4): if a site is DNase sensitive, the corresponding position is encoded as 1, otherwise 0.
- DNA methylation status (channel 5): similarly, the methylation status corresponds to a coding of 1, otherwise 0.

3. Functional area information (channels 6 and 7).

The important functional regions are labeled within the respective channels as follows:

- Protospacer and PAM region (channel 6): All positions within the protospacer and PAM region are labeled as 1.
- PBS region (channel 6): The entire PBS region is labeled as 1.
- RT region (channel 7): All positions within the RT region are labeled as 1.

The encoding approach described above was implemented to convert each input sequence (i.e. the Wild Target Sequence and the Edited Target Sequence) into a pseudo-image of a specific size. The dimensions of the pseudo-image correspond to the sequence length (128) and the number of channels (8). The pseudo-images were then aligned and spliced in the channel dimension to generate the input data, which had a size of 128 × 8 × 2. This method integrates sequence information, apparent information, and functional region features, while preserving the differences between the original and edited sequences. Consequently, it provides a more comprehensive feature representation for the model.

### Architecture of PrimeNet

The overall model architecture commences with the extraction of features via a multiscale convolution module (MixConv) [19], subsequently followed by a two-layer convolution operation for the purpose of further feature processing. The final stage of the architecture involves the spreading of features and their integration into a multibranch fully connected network (MLP) (Fig. 1b). The subsequent section provides a comprehensive delineation of the architecture.

#### Multiscale convolution module

In the context of predicting the efficiency of gene editing, the significance of features in DNA sequences varies at different scales. For specific sequence regions, localized features can influence editing efficiency, such as functional regions or mutation locations. In contrast, large-scale structural features of the sequence, such as the formation of a loop stem by the corresponding pegRNA, can play a pivotal role in other regions. Consequently, it is imperative to capture the features of DNA sequences at diverse spatial scales to ensure precise prediction of Prime editing efficiency.

For the purpose of extracting sequence features from multiple scales, the first layer of the model employs a multiscale convolution module (MixConv). This module performs convolution operations on the input data by using three different-sized convolution kernels (1 × 2, 3 × 2, and 9 × 2), respectively. These convolution kernels are designed to efficiently capture information at different scales [19]:

- Small-scale convolution (1 × 2): this convolution kernel is able to capture localized details in the sequence, such as apparent features in the PAM region. These small-scale features may play an auxiliary role in the prediction of editing efficiency.
- Mesoscale convolution (3 × 2): medium-sized convolution kernels allow better extraction of longer-range sequence features, capturing e.g. changes in functional regions or local upstream and downstream modulation information related to editing efficiency.
- Large-scale convolution (9 × 2): Large-scale convolution kernels enable the extraction of long-range dependencies within the sequence by covering a broader region. This capability is particularly crucial for capturing structural features such as the formation of a loop stem by the corresponding pegRNA, which plays a significant role in the dynamic process of gene editing.

This multibranch design retains the benefits of shared feature representations while concomitantly increasing the model’s flexibility and expressiveness via task-independent learning, yielding a modest yet consistent boost in overall performance.

To enhance feature representation, we applied Channel Attention [20] mechanism to the high-dimensional feature maps extracted by multiscale convolution, allowing the model to focus on key feature dimensions and adjust weights accordingly. We then used a 1 × 1 convolution layer to compress these features, reducing dimensionality and mitigating overfitting while preserving essential information.

#### Attention mechanism

This model integrates two attention mechanisms: Channel-attention [20] and Conv-attention [21]. Given that both the channel features and spatial location information of the input data are critical for the prediction task, we introduce Channel-attention and Conv-attention accordingly. Channel-attention is designed to emphasize channel-wise feature importance and is applied after the multiscale convolutional layer, before feature propagation into the multibranch fully connected network (MLP), ensuring effective weighting of channel information. In contrast, Conv-attention focuses on spatial location information and is incorporated both before the input data enters the model and after each convolutional layer to enhance spatial feature extraction. The detailed implementation of these two attention mechanisms is described below.

#### Channel Attention

In order to prioritize the channel attributes of pseudo-images, this model incorporates the Channel Attention mechanism proposed by Wang *et al*. [20] to enhance the model’s capacity for feature selection and information representation. The fundamental principle of the Channel Attention mechanism is to adaptively allocate weights to each channel, thereby enabling the model to prioritize the most salient feature channels while suppressing redundant or less significant channels. This enhances the model’s capability to discern key features.

Specifically, a channel attention mechanism is designed based on global average pooling (Fig. 1c). This mechanism generates the attention weights of each channel by performing adaptive pooling and convolution operations on the input feature map. It then performs element-by-element weighted combination with the input feature map.

##### Conv-attention

To capture the spatial characteristics of pseudo-images, we employed the Conv-attention mechanism [21] with convolutional operations to generate adaptive spatial weights. This facilitates the model’s ability to emphasize important regions and suppress unimportant ones, thereby enhancing the accuracy of feature representation. In contrast to conventional softmax activation, we utilized sigmoid activation to enable independent weight adjustment for each spatial location. This modification empowers the model to discern nuanced patterns and prioritize relevant features. Softmax activation is less suitable for tasks with varying spatial importance, whereas sigmoid activation provides a more flexible way to weight different regions [22].

We conducted ablation experimental studies to investigate the efficacy of different activation functions and convolution kernel size in the model. Specifically, we examined the performance of the model with sigmoid and softmax for activation functions, as well as kernel size of {1,3,5,7,9,11}. The result of our study, detailed in the supplementary information, demonstrate that the Conv-attention mechanism with Sigmoid activation function and kernel size of 5 enhances the model’s performance (Supplementary Figs. S2, S3).

#### Multibranch MLP

In this model, a multibranch fully connected network is employed in the final layer. The fundamental principle underlying this design is to allocate independent parameter update space for each prediction target (i.e. validly edited, unedited, and erroneously edited) to circumvent feature interference and negative interactions between different prediction targets.

In standard multilayer perceptron (MLP), all tasks share the same set of network parameters. This design may lead to feature interactions between different goals, which in turn may exhibit conflicts or this-and-that phenomenon between features on the validation set [23, 24]. To address this issue, we draw inspiration from the approaches of Ando and Zhang [25] as well as Blitzer *et al*. [26]. We propose a methodology that involves the establishment of independent branches for each prediction goal, with each branch possessing exclusive weights and parameters. This approach allows each branch to focus on learning the features most relevant to its specific objective. Specifically, the model extracts common features from the input data through the shared part of the MLP, thereby ensuring that all prediction targets share a uniform base feature representation. The shared part is primarily responsible for preliminary processing and feature extraction of input features, thereby providing consistent base information for each task. Subsequently, the branching part of each task independently learns specific features related to its target. This design ensures that each prediction target can adjust its weights according to its specific needs, avoiding interference between different tasks and thus improving the prediction accuracy of each efficiency value.

This multibranch design not only retains the advantages of shared features, but also enhances the flexibility and expressiveness of the model through task-independent learning, which ultimately improves the overall performance effectively.

### Training scheme

The HEK293T (107 165 samples) and K562 (18 742 samples) datasets were partitioned into training, validation, and test sets at an 8:1:1 ratio. The two training subsets were subsequently integrated, randomly rearranged, and employed to train the model. In the training process of this model, various strategies were employed to enhance its stability and efficacy, including weight initialization methods and optimization algorithms.

#### Orthogonal weight initialization

To ensure the training stability of the model and accelerate the convergence, we adopt the orthogonal initialization method [27]. By making the weight matrix satisfy orthogonality, this method can effectively avoid the problem of disappearing or exploding gradient at the early stage of training, thus improving the training stability of the model.

Orthogonal initialization constitutes a pivotal component in the training of deep neural networks, as it plays a crucial role in ensuring the stability of the gradient flow at each layer of the network. This is achieved by guaranteeing that the rows or columns of the weight matrix are mutually perpendicular. To elaborate, for the weight matrices W associated with the convolutional and fully connected layers, the initialization process is meticulously designed to ensure that they satisfy the following conditions:


$$ {\mathrm{W}}^{\mathrm{T}}\mathrm{W}=\mathrm{I} $$

where I is a unit matrix indicating that the column vectors of the weight matrix W are orthogonal. Orthogonal initialization has been shown to improve training stability by ensuring that the gradient does not disappear or explode rapidly due to the effect of the product chain during forward and backward propagation. In addition, for the bias term *b*, zero initialization is employed to avoid the influence of the bias term on the network learning in the early stage and to maintain the initial state of the network as simple as possible.

#### Lookahead optimization

In order to enhance the optimization effect and expedite the model convergence, the Lookahead method, as proposed by Zhang *et al*. [28], is hereby introduced. This method maintains two distinct sets of parameters: the slow weights, denoted by φ, and the fast weights, denoted by θ. At the beginning of each outer iteration *t*, the fast weights are synchronized with the slow weights by setting ${\theta}_{t,0}\leftarrow{\varphi}_{t-1}$. Then, for a fixed number *k* of inner iterations, the fast weights are updated using any standard optimizer *A* (such as SGD or Adam). Specifically, for each inner step *i* (where *i* = 1, 2, …, *k*), a mini-batch *d* is sampled from the dataset *D* and the fast weights are updated according to the rule:


$$ {\theta}_{t,i}={\theta}_{t,i-1}+A\left(L,{\theta}_{t,i-1,}d\right) $$

In this expression, *L* represents the loss function being minimized, and $A\left(L,{\theta}_{t,i-1,}d\right)$ computes the update (typically involving the gradient $\nabla \theta L$) for the current fast weights ${\theta}_{t,i-1}$ based on the sampled mini-batch *d*. After completing these *k* inner updates, the slow weights are updated by moving them a fraction α (with 0 < α ≤ 1) toward the final fast weights ${\theta}_{t,k}$ using the interpolation


$$ {\varphi}_t={\varphi}_{t-1}+\alpha \left({\theta}_{t,k}-{\varphi}_{t-1}\right) $$

Here, α is the slow weight step size that controls the extent to which the fast weights influence the slow weights, effectively implementing an exponential moving average that reduces the variance inherent in the inner updates. Finally, to ensure stability, the fast weights are reset to the newly updated slow weights (i.e. ${\theta}_{t,0}$ is set equal to ${\varphi}_t$) before the next outer iteration begins. In summary, ${\varphi}_t$ represents the slow weights at iteration *t*, ${\theta}_{t,i}$ denotes the fast weights after the *i*-th inner update within the *t*-th outer loop, *k* is the number of inner updates performed per outer iteration, *d* is a mini-batch sampled from *D*, and *A*(*L*,*θ*,*d*) is the update provided by the inner optimizer based on the current parameter values, loss function *L*, and mini-batch *d*. By decoupling the rapid, exploratory updates (fast weights) from the stable, long-term trajectory (slow weights), Lookahead effectively combines the advantages of aggressive search and variance reduction, leading to more robust and accelerated convergence in practice. When combined with the orthogonal weight initialization, the model performs smoothly during the training process (Supplementary Fig. S4).

#### Bayesian hyperparameter tuning

We employ a Bayesian optimization approach to calibrate the hyperparameters of the model to enhance the efficacy of the model. Specifically, the Optuna library [29] was utilized for the automated search of hyperparameters to achieve optimal model results. Optuna is an efficient hyperparameter optimization framework that progressively approximates the optimal hyperparameter combinations based on historical experimental results through Bayesian optimization strategies.

In our case, hyperparameter optimization was performed to maximize Spearman correlation of validly edited efficiencies, which automatically tuned parameters including convolutional channel sizes, learning rate, and attention mechanism configurations. A multibranch attention framework was implemented, allowing selective activation of Conv-attention and Channel Attention modules to optimize performance. The full hyperparameter search space and sampling strategies are summarized in Supplementary Table S1, and the best-performing configuration selected by Optuna is listed in Supplementary Table S2.

## Results

### PrimeNet outperforms existing Prime editing efficacy prediction models

A systematic comparison was conducted among the PRIDICT family of models [9, 16], DeepPE [8], DeepPrime [10], and our PrimeNet model. The evaluation was carried out in two settings: one using the original weights provided by the respective authors (“original”) and the other retraining the models from scratch (“retrained”).

First, our model, PrimeNet, demonstrated robust performance across both cell lines. In the HEK293T dataset, the values of Spearman correlation coefficient and Pearson correlation coefficient were 0.94/0.94 for samples that were validly edited, 0.93/0.94 for samples that were not edited, and 0.85/0.85 for samples that were erroneously edited. In the K562 dataset, the model reached 0.82/0.91 for valid edits, 0.81/0.88 for unedited outcomes, and 0.51/0.63 for erroneous edits.

In the context of the original-weight setting, the PRIDICT model demonstrated its applicability exclusively to HEK293T, yielding Spearman/Pearson correlation coefficients of 0.82/0.84, 0.79/0.82, and 0.47/0.44 for the three categories. The original PRIDICT2 model demonstrated superior performance, with 0.87/0.88, 0.84/0.86, and 0.66/0.66 on HEK293T dataset, and 0.76/0.63, 0.80/0.66, and 0.71/0.66 on K562 dataset. It is noteworthy that the DeepPE model was initially developed to process only 47-bp sequences and to predict valid editing outcomes. However, it exhibited a substantial mismatch with the 99-bp input sequences, resulting in significantly diminished performance on HEK293T, with only 0.13/0.11 achieved.

As to the context of the retrained setting, PRIDICT2 attained Spearman/Pearson correlation coefficients of 0.82/0.82, 0.85/0.85, and 0.78/0.75, respectively, for valid, unedited, and erroneous samples, utilizing the HEK293T dataset to retrain the model. For the K562 cell line, the recorded values were 0.72/0.53, 0.65/0.55, and 0.22/0.32, where a substantial decrease was observed in the unedited category. Following a comprehensive retraining procedure, DeepPE attained 0.55/0.56, 0.62/0.64, and 0.61/0.53 on the HEK293T dataset, as well as 0.51/0.29, 0.46/0.33, and 0.20/0.25 on the K562 dataset. These results indicate that the model possesses the capacity to generate viable predictions even when applied to 99-bp sequences. DeepPrime demonstrated consistent performance across various datasets, achieving 0.86/0.86, 0.89/0.89, and 0.80/0.78 on HEK293T dataset, as well as 0.70/0.55, 0.63/0.56, and 0.21/0.32 on K562 dataset.

**Figure 2:**
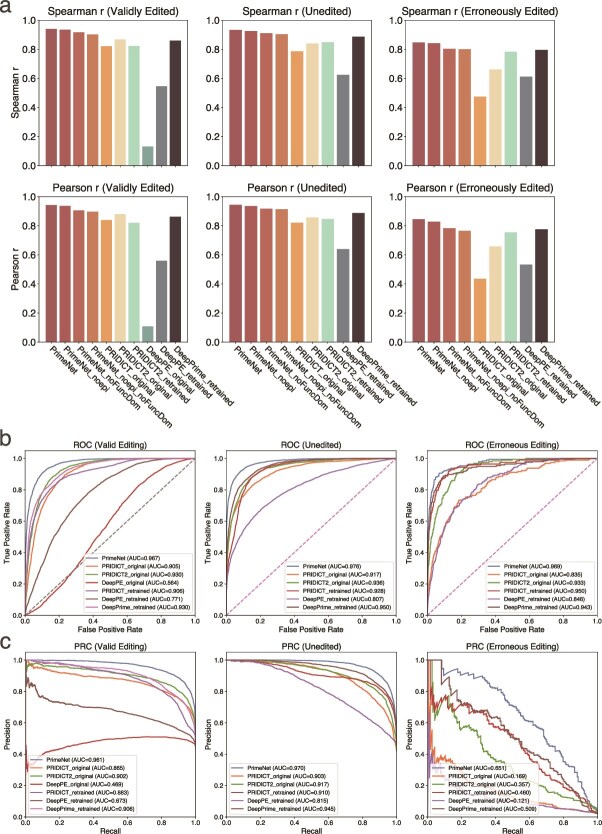
Comparison of model performance on HEK293T dataset. **a** Spearman *R* and Pearson *r* values for all models. (Note: the *x*-axis labels for the Spearman *R* plot are identical to those in the Pearson *r* plot.) **b** ROC curves for all models, illustrating that the PrimeNet model achieves the highest true-positive rate relative to its false-positive rate. **c** PR curves for all models, further confirming that the PrimeNet model exhibits the best performance in terms of both precision and recall.

**Figure 3:**
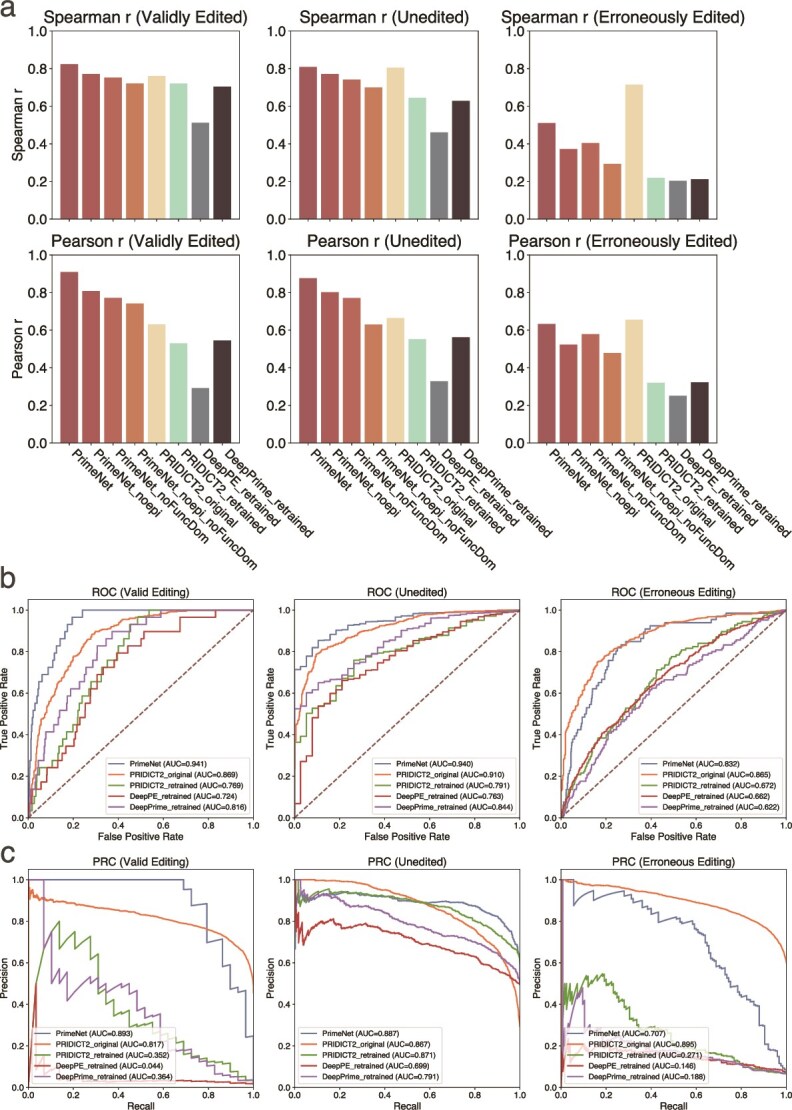
Comparison of model performance on K562 dataset. **a** Spearman *R* and Pearson *r* values for all models. (Note: the *x*-axis labels for the Spearman *R* plot are identical to those in the Pearson *r* plot.) **b** ROC curves for all models, illustrating that the PrimeNet model achieves the highest true-positive rate relative to its false-positive rate. **c** PR curves for all models, further confirming that the PrimeNet model exhibits the best performance in terms of both precision and recall.

The PrimeNet model attains a comprehensive breakthrough through an innovative architecture and the incorporation of epistatic data, the predictive performance of which is demonstrated in Fig. 2 and Fig. 3. The performance results of the PRIDICT family of models as well as DeepPE on the PrimeNet independent test set are presented in Fig. 2 and Fig. 3. In addition, Fig. 2 and Fig. 3 shows the performance metrics of three models that use the PrimeNet architecture but do not fully incorporate the epistemic and functional domain information. Notably, the model exhibits a consistent performance superiority over PRIDICT, even in the absence of domain information. This outcome underscores the efficacy of the architecture employed by PrimeNet, which integrates Convolutional Neural Networks with MLP, augmented by an attention mechanism. In comparison, the bidirectional attention-based Recurrent Neural Networks architecture utilized by PRIDICT in this particular task demonstrates a comparatively lower performance. It should be noted that the original DeepPrime model was not included in this standard test set comparison due to its limitation of supporting only ≤3 base edits.

To comprehensively evaluate the performance of each model in the three tasks of effective editing, non-editing, and incorrect editing, we uniformly converted the continuous predictors corresponding to each task into binary labels and adopted 50% as the judgment threshold. That is, for all tasks, if the predicted or true value is >50%, it is judged as a positive case (indicating a higher probability of occurrence), and vice versa as a negative case. Since the DeepPE model only predicts valid edits, its results are not included in the unedited and incorrectly edited tasks. Based on this dichotomous classification strategy, we computed ROC curves and precision–recall (PR) curves for each model under different tasks and used the area under the curve (AUC) as a performance measure. To visually compare the performance of each model in the three tasks, we plot the ROC curves and PR curves for effective editing, no editing, and incorrect editing in Figs. 2b, 2c, 3b, and  3c, respectively. The experimental results show that the PrimeNet model exhibits optimal performance in all tasks: its ROC curve is obviously closer to the upper left corner and has the highest AUC value; and on the PR curve, PrimeNet maintains a high precision rate even at a high recall rate. These results fully prove that the PrimeNet model has excellent discriminative ability and robustness in the three tasks of effective editing, non-editing, and false editing, thus demonstrating its high practicality and robustness in practical applications.

### Effect of epigenetic modifications on editing efficiency in Prime editing

In order to assess the effect of epigenetic modifications on the editing efficiency of Prime editing, statistical analyses were performed on chromatin openness and methylation levels. First, chromatin openness was categorized into two groups, “completely open” and “presence of closure,” and then the Mann–Whitney *U* test was performed for three types of editing efficiencies, including valid editing efficiency, unedited efficiency, and erroneous editing efficiency. The results indicated that chromatin openness exhibited a substantial impact on the three efficiency values of Prime editing, with all *P*-values <.001.

Similarly, methylation levels were categorized into two groups, with and without methylation, respectively. The Mann–Whitney *U* test was then performed on each of the three efficiency values. The results of this analysis indicated that methylation status significantly affected the editing efficiency of Prime editing, with *P*-values <.001.

**Figure 4:**
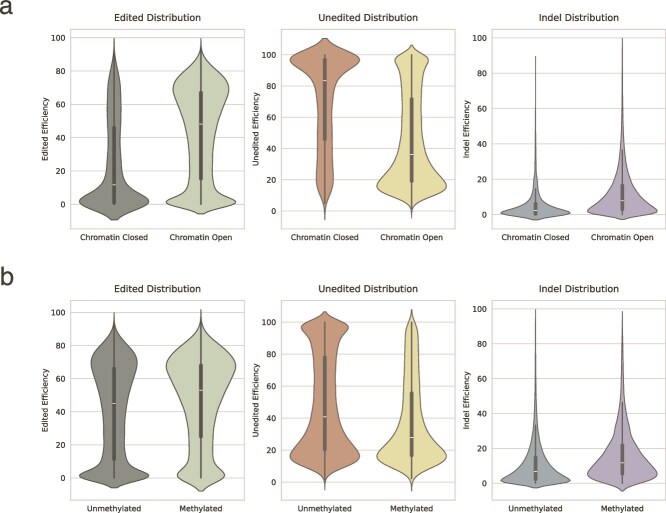
Impact of epigenetic modifications on prime editing efficiency. **a** Effect of chromatin accessibility on Prime editing efficiency, with *P*-values <.001. **b** Effect of DNA methylation on Prime editing efficiency, with *P*-values <.001.

In summary, both chromatin openness and methylation levels have been demonstrated to significantly affect the editing efficiency of Prime editing. To further visualize this result, the relationship between chromatin openness and methylation levels and the three editing efficiencies was visualized by means of violin plots (see Fig. 4a and 4b).

### PrimeNet provides multi-perspective interpretability as an interpretable AI model

#### General feature attribution from instance study

The saliency map is employed to demonstrate the extent to which each input feature influences the model’s predictions. Generated using the Integrated Gradients method from the Captum library [30], it is designed to elucidate the basis of the model’s decisions in different tasks (validly edited, unedited, and erroneously edited).

In this study, we applied Integrated Gradients to a sample derived from HEK293T and harboring the pathogenic GJB2 variant NM_004004.6(GJB2):c.269. The T > C (p. Leu90Pro) mutation was created through the implementation of Prime editing, which converted the reference A to G. The design of the pegRNA, encompassing a 13-nt primer-binding site, a 15-nt reverse-transcriptase extension, and a 20-nt template, positioned the edit four nucleotides upstream of the PAM on the forward strand. Deep sequencing of this sample yielded a validly editing efficiency of 35.11%, an unedited fraction of 38.86%, and erroneous editing products (including indels and off-target substitutions) at 26.03%, which are largely in compliance with the prediction result of our PrimerNet (validly edited of 33.36%, unedited of 42.9%, and erroneously edited of 23.74%). We then generate saliency maps and assess the relative importance of various input features, including gene sequence context, DNase accessibility, and methylation status in predicting Prime editing efficiency, as well as to elucidate why this particular sample exhibited these editing efficiencies in order to inform potential improvements.

**Figure 5:**
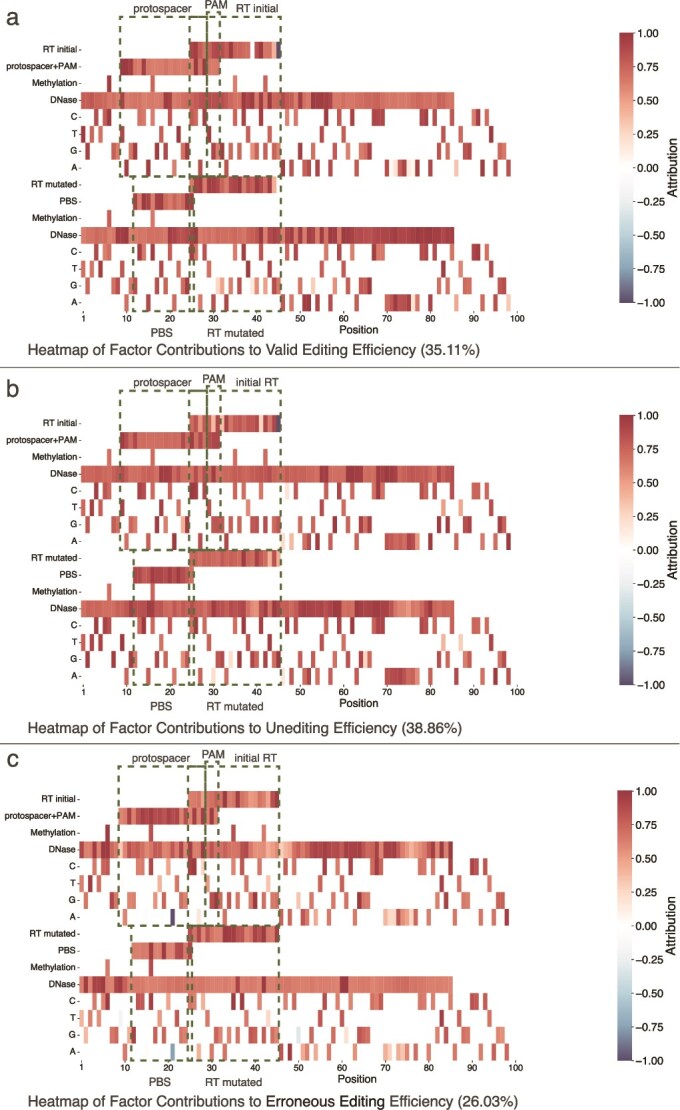
General feature attribution significance heatmap. **a** Attribution heatmap for effective editing efficiency (observed efficiency: 35.11%). The saliency map analysis identifies key factors influencing editing efficiency, with areas of high attribution representing positive regulators that promote editing and areas of low attribution indicating negative regulators that inhibit editing. The heatmap quantifies the contribution of genomic features, providing insights into the regulatory basis of the 35.11% observed efficiency. **b** Attribution heatmap for unedited efficiency (observed efficiency: 38.86%). Feature attribution analysis highlights key factors maintaining the unedited state (high attribution) and features promoting editing (low attribution). The heatmap quantifies the contributions of these factors, explaining the 38.86% observed unedited efficiency. **c** Attribution heatmap for erroneous editing efficiency (observed efficiency: 26.03%). Sequence features driving erroneous editing are marked with high attribution, while biomarkers suppressing unintended modifications are marked with low attribution. The heatmap quantifies the contribution of these regulatory elements, elucidating the mechanisms underlying the 26.03% observed erroneous editing efficiency.

The utilization of saliency maps (Fig. 5) facilitates a more profound comprehension of the model’s decision-making process. The reddish regions of the heatmap signify a high positive effect on the prediction, suggesting a favorable association with gene editing. Conversely, the bluish regions indicate a negative effect on the prediction, potentially implicating a correlation with diminished editing efficiency. Consequently, the model unveils the specific sequence regions or biomarkers that exert the most substantial influence on the decision-making process when making validly edited, unedited, and erroneously edited predictions. This knowledge serves as a valuable reference for gene editing researchers, particularly in the context of designing gene editing tools such as Prime editing. It facilitates the optimization of editing strategies, the identification of pivotal regions, and the refinement of experimental design, thereby enhancing the efficacy of gene editing.

#### Epigenetic feature attribution

To thoroughly investigate the contributions of chromatin accessibility (channel 4) and DNA methylation (channel 5) in model prediction, a gradient ascent-based optimization method was devised with the objective of maximizing the value of the target output (i.e. the editing efficiency) to generate the feature representations of these two channels. Initialization of the input image and activation of channels 4 and 5, respectively, occurred with masking of the gradients of the other channels to ensure that only the effect of the target channel on the editing efficiency was considered in the optimization process. To avoid uncertainty in the optimization results due to the poor performance of the features of other channels, the top 200 sequences with the highest editing efficiency were selected for optimization. Finally, to obtain a general and representative feature representation, the optimization results were averaged to circumvent the idiosyncratic effect of a single sample.

##### Chromatin accessibility

**Figure 6:**
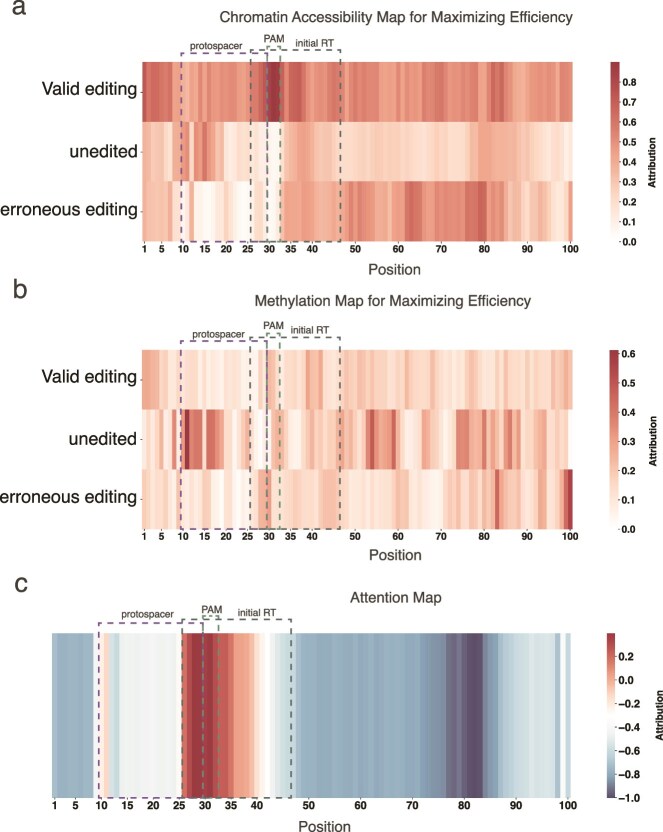
Epigenetic feature attribution and model attention distribution. **a** Visualization of chromatin accessibility, illustrating the conditions under which chromatin accessibility promotes the corresponding efficiency. **b** Visualization of DNA methylation, demonstrating how different DNA methylation states influence efficiency. **c** Attention distribution visualization of the model, highlighting the regions that the model primarily focuses on during prediction.

As demonstrated in Fig. 6a, values approaching 1 signify chromatin accessibility, while values approaching 0 indicate inaccessibility. It is noteworthy that the observed chromatin accessibility may exhibit substantial variation depending on the optimization target. When the protospacer and PAM regions exhibit high openness, the binding capability of pegRNA is augmented, thereby facilitating the Cas9 protein’s recognition of the PAM region and enhancing gene editing efficiency. Conversely, when the protospacer and PAM regions remain closed, particularly when the PAM region is fully closed, Cas9 protein binding is inhibited, which, in turn, reduces the initiation of the editing process and decreases the frequency of gene editing events. If the RT binding region (initial RT) remains open and other regions remain closed, this can lead to an elevated probability of erroneous editing.

##### DNA methylation

In Fig. 6b, a value close to 1 indicates that the site is methylated, while a value close to 0 indicates that the site is unmethylated. As indicated by the literature, DNA methylation, especially on CpG islands, is a key factor in the regulation of gene expression. Methylation usually affects the structure of DNA, making it more compact and inhibiting the binding of transcription factors and other regulators to DNA, thereby reducing the transcriptional activity of genes [31, 32]. In the context of Prime editing, the methylation status of the target site exerts a significant influence on the binding efficiency of pegRNA and the recognition of the PAM region by Cas9 proteins [15]. This is due to the fact that methylated DNA typically exhibits reduced accessibility, which impacts these processes.

It has been demonstrated that the methylation levels of the protospacer and PAM regions are critical factors in the gene editing process. When these regions exhibit low methylation levels, the chromatin structure becomes unraveled, thereby facilitating the binding of pegRNA to the target DNA and enhancing the recognition of the PAM region by the Cas9 protein. This, in turn, promotes the efficiency of gene editing. Conversely, elevated methylation levels in the protospacer and PAM regions result in a more compact chromatin structure, impeding pegRNA binding and Cas9 protein recognition, and consequently reducing the frequency of gene editing events. Notably, demethylation of the protospacer region and moderate methylation levels in the PAM region can potentially diminish editing precision, potentially leading to erroneous editing outcomes.

##### Histone modification

In addition, we investigated the incorporation of H3K4me3 ChIP-seq data to assess its capacity of enhancing model performance. The findings indicated that the incorporation of H3K4me3 features resulted in a modest enhancement of ~0.001 in the Spearman correlation coefficient (Supplementary Fig. S5). Conversely, DNA methylation (RRBS) and chromatin accessibility (DNase-seq) features have already yielded sufficiently comprehensive and stable epigenomic information. Furthermore, the acquisition of histone modification data is associated with higher costs, greater variability in signal-to-noise ratios across batches, and the necessity of complex preprocessing workflows that are challenging to standardize across diverse platforms. This, in turn, can lead to a heightened risk of compromised data integrity and consistency [33]. Given the objective of this study, which is to develop an efficient, robust, and broadly applicable prediction framework across multiple cell lines, we ultimately elected to focus on DNA methylation and chromatin accessibility features, foregoing further inclusion of histone modification data. This decision was made to streamline the data-processing pipeline, reduce computational resource demands, and ensure the model’s reliability and reproducibility in practical applications.

#### Attention map

The model’s attention weights were extracted to construct an Attention Map (Fig. 6c), which aims to reveal the regions that the model focuses on when processing the input data. The Attention Map visualizes the distribution of the model’s attention at different spatial locations. As shown in Fig. 6c, the results of the Attention Map clearly indicate that the model’s attention is focused on key regions such as the protospacer region, the PAM region, and the RT region. These regions have been identified as playing a pivotal role in the Prime editing process, suggesting that the model is capable of effectively focusing on important sequence regions related to editing efficiency when making predictions.

## Discussion

PrimeNet effectively addresses the key limitations of existing models by integrating epigenetic factors and utilizing deep learning architectures. The findings demonstrate that PrimeNet attains a state-of-the-art level of proficiency in predicting valid editing efficiency, unedited efficiency, and erroneous editing efficiency, as evidenced by Spearman correlation coefficients of 0.94, 0.93, and 0.85 for HEK293T dataset as well as 0.82, 0.81, and 0.51 for K562 dataset, respectively. This enhancement in performance underscores the significance of incorporating data on chromatin accessibility and DNA methylation into the prediction framework, as these factors exert a substantial influence on the binding efficiency of pegRNA and the accessibility of target DNA regions. It is noteworthy that the integration of multiscale convolutional layers with the attention mechanism enables PrimeNet to capture both local sequence features and long-range dependencies, which is crucial for modeling complex interactions in gene editing systems.

A pivotal finding of this study is the substantial impact of chromatin accessibility and DNA methylation on Prime editing outcomes. Statistical analysis revealed that open chromatin or hypomethylated regions considerably augmented editing efficiency, which can be ascribed to the enhanced binding efficacy of the pegRNA–Cas9 complex and the mitigation of spatial site resistance. Conversely, closed chromatin or hypermethylated states in protospacer and PAM regions were associated with reduced editing activity, which is consistent with the observation that epigenetic states modulate CRISPR editing tools in previous studies. These findings underscore the necessity to incorporate epigenetic data into predictive models, as neglecting such factors may lead to overestimation of editing efficiency in heterochromatin-enriched regions or methylated sites. Furthermore, interpretability analysis based on significance maps and gradient optimization showed that PrimeNet is able to dynamically resolve the contribution weights of epigenetic features (e.g. chromatin accessibility versus methylation status) to editing efficiency and visually present the epigenetic modification patterns of key functional regions (e.g. protospacer versus PAM regions). Additionally, PrimeNet’s interpretability framework surpasses conventional “black-box” models by revealing feature contributions through saliency maps and attention weights. This transparency not only validates the biological relevance of model predictions but also enables researchers to prioritize target regions or epigenetic modifiers in experimental design.

Additionally, large pretrained DNA models (e.g. Evo2, Generator) [34, 35] exhibited suboptimal performance in predicting Prime editing outcomes when compared to purpose-built architectures (Supplementary Table S3). This deficiency can be attributed to the fact that these models are capable of capturing broad sequence statistics; however, they lack the mechanistic understanding of local DNA repair, guide RNA binding, and nucleotide modifications that are crucial for Prime editing. Models that have been specifically trained on Prime editing data, incorporating both sequence context and repair enzyme kinetics, are better suited for this task. Subsequent endeavors should prioritize the construction or refinement of extensive models directly on Prime editing datasets, incorporating biochemical knowledge and attention mechanisms customized to the editing process.

Despite the significant advancements made, it is imperative to acknowledge the limitations of PrimeNet, which require further exploration. Primarily, the model’s training was based on HEK293T and K562 cell line data, and its capacity to generalize to other cell types, such as neurons or stem cells, remains to be validated. The variability of epigenetic landscapes across tissues is a notable concern, and future studies should prioritize the validation of PrimeNet’s applicability with diverse cell line or *in vivo* model datasets. Second, while the current study prioritized the analysis of chromatin accessibility and DNA methylation, the consideration of additional epigenetic regulators, such as histone modifications or three-dimensional chromatin structure, has been omitted. The incorporation of these factors into the feature space may lead to a further enhancement in prediction accuracy. Finally, the validation of the prediction results of PrimeNet by high-throughput experiments is necessary to ascertain its practical usefulness in real-world scenarios. These can further improve the model’s understanding of the data from the ground up, thus further reducing the computational complexity of the model and achieving the goal of optimizing computational efficiency.

PrimeNet demonstrates the transformative potential of integrating deep learning with epigenetic insights in precision genome engineering. By offering computationally efficient and interpretable tools, this study facilitates the transition from trial-and-error pegRNA design to data-driven strategies. In the therapeutic domain, PrimeNet has the potential to streamline the development process for gene therapies by mitigating off-target effects and optimizing target activity, particularly in the context of treating epigenetically complex diseases such as cancer and neurodegenerative disorders. Moreover, the framework delineated in this study could serve as a foundation for the development of analogous approaches for other CRISPR-derived editors, thereby propelling the advancement of next-generation predictive models that integrate sequence, structure, and epigenetics.

## Conclusion

PrimeNet implements a paradigm shift in the prediction of Prime editing efficiency, thereby bridging the gap between computational modeling and biological complexity. Its integration of multi-omics data, advanced neural architectures, and interpretable outputs renders it a versatile tool for both basic research and translational applications. Future iterations of PrimeNet, optimized by expanding the dataset and adding new epigenetic layers, are expected to unlock the full potential of Prime editing in genome engineering.

**Key Points**

- PrimeNet demonstrates exceptional performance, achieving a Spearman correlation coefficient of 0.94 on HEK293T dataset and 0.82 on K562 dataset, respectively, indicating a very strong positive correlation between its prediction results and actual editing efficiency.
- By incorporating data from multiple cell lines and introducing multiscale convolution and attention mechanisms, PrimeNet can capture different sequence features and generalize well for unseen cell lines. This enables researchers to more effectively guide experimental design, enhance the success rate of gene editing, and reduce unnecessary experimental costs.
- The integration of significant epigenetic factors, including chromatin accessibility and DNA methylation, enables PrimeNet to more comprehensively grasp the intricate mechanisms underlying gene editing across diverse cell lines, thereby enhancing the accuracy of predictions.

## Supplementary Material

revised_Supplementary_material_bbaf293

## Data Availability

The model and model usage are available on the GitHub at https://github.com/bm2-lab/PrimeNet.
