# Supplementary material for: PrimeNet: rational design of Prime editing pegRNAs by deep learning
Source: Brief Bioinform. 2025 Jun 19;26(3):bbaf293. doi: 10.1093/bib/bbaf293 (PMC12204610; doi:10.1093/bib/bbaf293)
Supplement: revised_Supplementary_material_bbaf293 [file revised_supplementary_material_bbaf293.docx]

**1. Distribution of PRIDICT dataset**


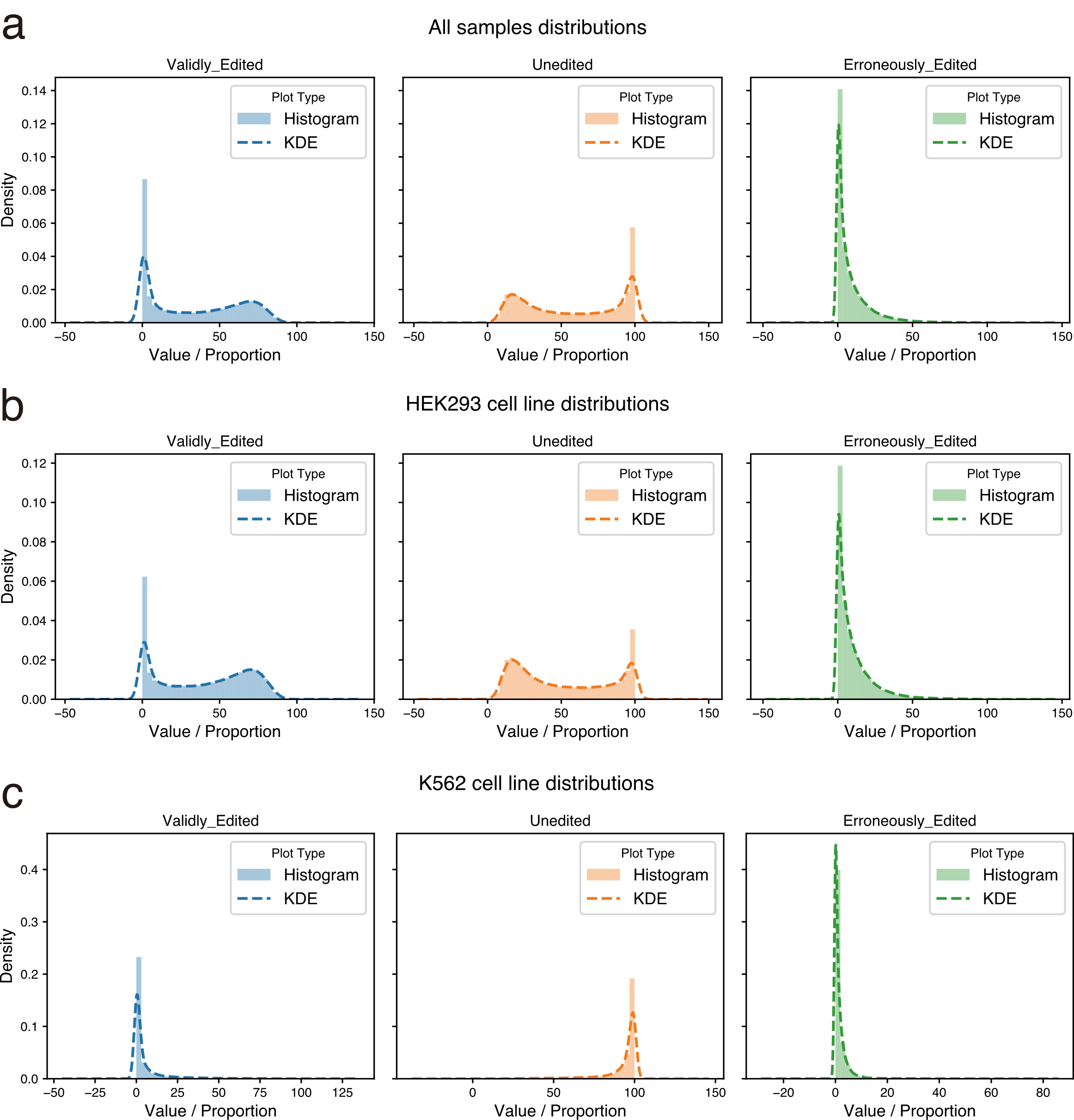


**Fig. S1** Data distribution plots. **a** Distribution for all samples. **b** Distribution for the HEK293 cell line. **c** Distribution for the K562 cell line.

2. Ablation study on Sigmoid and Softmax activation function

The efficacy of the Sigmoid activation function in the Conv-Attention mechanism was assessed by comparing the performance of models utilizing Sigmoid and Softmax activation functions. As illustrated in Figure a, the experimental findings demonstrate that the model employing Sigmoid activation function demonstrates a substantial enhancement in performance in comparison to the model utilizing Softmax activation function in the context of Prime editing efficiency prediction. In terms of validly edited efficiency, the PrimeNet model employing the Sigmoid activation function attained a Spearman correlation coefficient of 0.94 and a Pearson correlation coefficient of 0.95. Conversely, the PrimeNet model utilizing the Softmax activation function achieved a Spearman correlation coefficient of 0.80 and a Pearson correlation coefficient of 0.81, respectively. In terms of unedited efficiency, the PrimeNet model employing the Sigmoid activation function attained a Spearman's correlation coefficient of 0.94 and a Pearson's correlation coefficient of 0.95, which were considerably higher than the model utilizing the Softmax activation function (Spearman's correlation coefficient 0.81 and Pearson correlation coefficient 0.82). With regard to erroneous edited efficiency, the PrimeNet model employing the Sigmoid activation function yielded a Spearman correlation coefficient of 0.85 and a Pearson correlation coefficient of 0.85, while the model utilizing the Softmax activation function exhibited a Spearman correlation coefficient of 0.70 and a Pearson correlation coefficient of 0.66.

In addition, a comparison was made of the absolute errors of model predictions under two different activation functions (Sigmoid and Softmax). The KDE (Kernel Density Estimation) of the absolute error for each target variable (validly edited, unedited, and erroneously edited) was plotted (Fig. b), and it was found that the model using the Softmax activation function exhibited a significantly larger distribution of absolute errors on all three target variables. This finding indicates that the Softmax activation function tends to bias predictions more significantly than the Sigmoid activation function when utilizing the same task settings.

Specifically, the error distributions of the Softmax activation functions are significantly wider, especially with a higher frequency in the larger error ranges. This finding indicates that the Softmax model exhibits elevated prediction uncertainty on these specific tasks, which may result in increased misclassification or prediction errors. In contrast, the model employing the Sigmoid activation function demonstrates a comparatively small and centralized error distribution, suggesting that it performs with greater consistency and accuracy in prediction tasks.


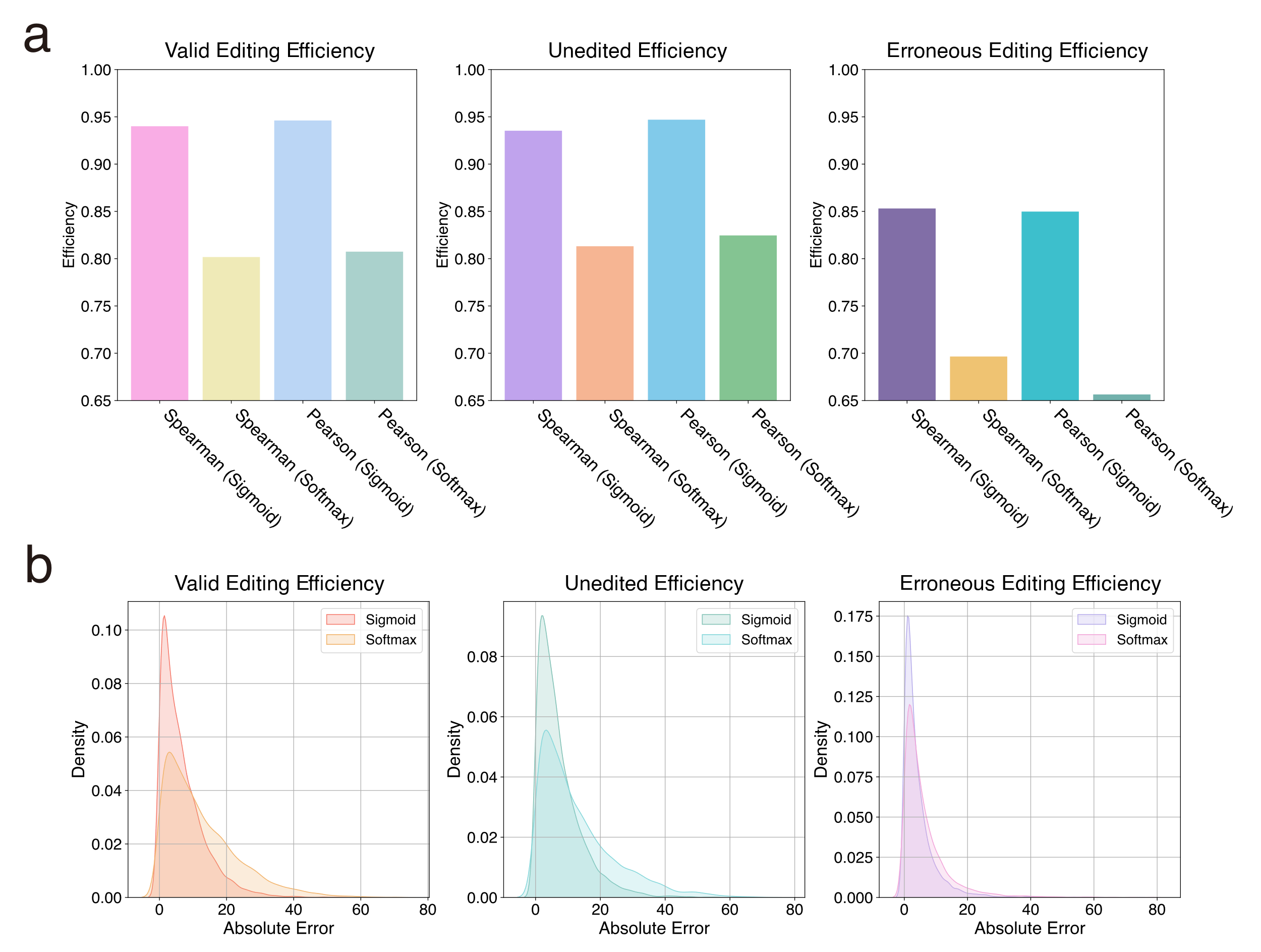


**Fig. S2** Performance comparison of PrimeNet models using Sigmoid and Softmax activation functions. **a** Spearman and Pearson correlation coefficients for validly edited, unedited, and erroneously edited efficiencies, demonstrating the superior performance of the Sigmoid activation function over Softmax in Prime editing efficiency prediction. **b** Kernel Density Estimation (KDE) of absolute errors for the three target variables, showing that the Softmax-based model exhibits a wider error distribution, indicating higher prediction uncertainty compared to the Sigmoid-based model.

### 3. Ablation study on convolution kernel size in Conv-Attention

To evaluate the impact of convolution kernel size in our Conv-Attention architecture, we conducted an ablation study varying the kernel size (k) across multiple values. The results, visualized in the boxplot (Figure S3), show that although performance fluctuates slightly with different kernel sizes, k = 5 consistently yields a higher median Spearman R and lower variance across trials, indicating more stable and optimal performance. Therefore, we select k = 5 as the default configuration in our final model.


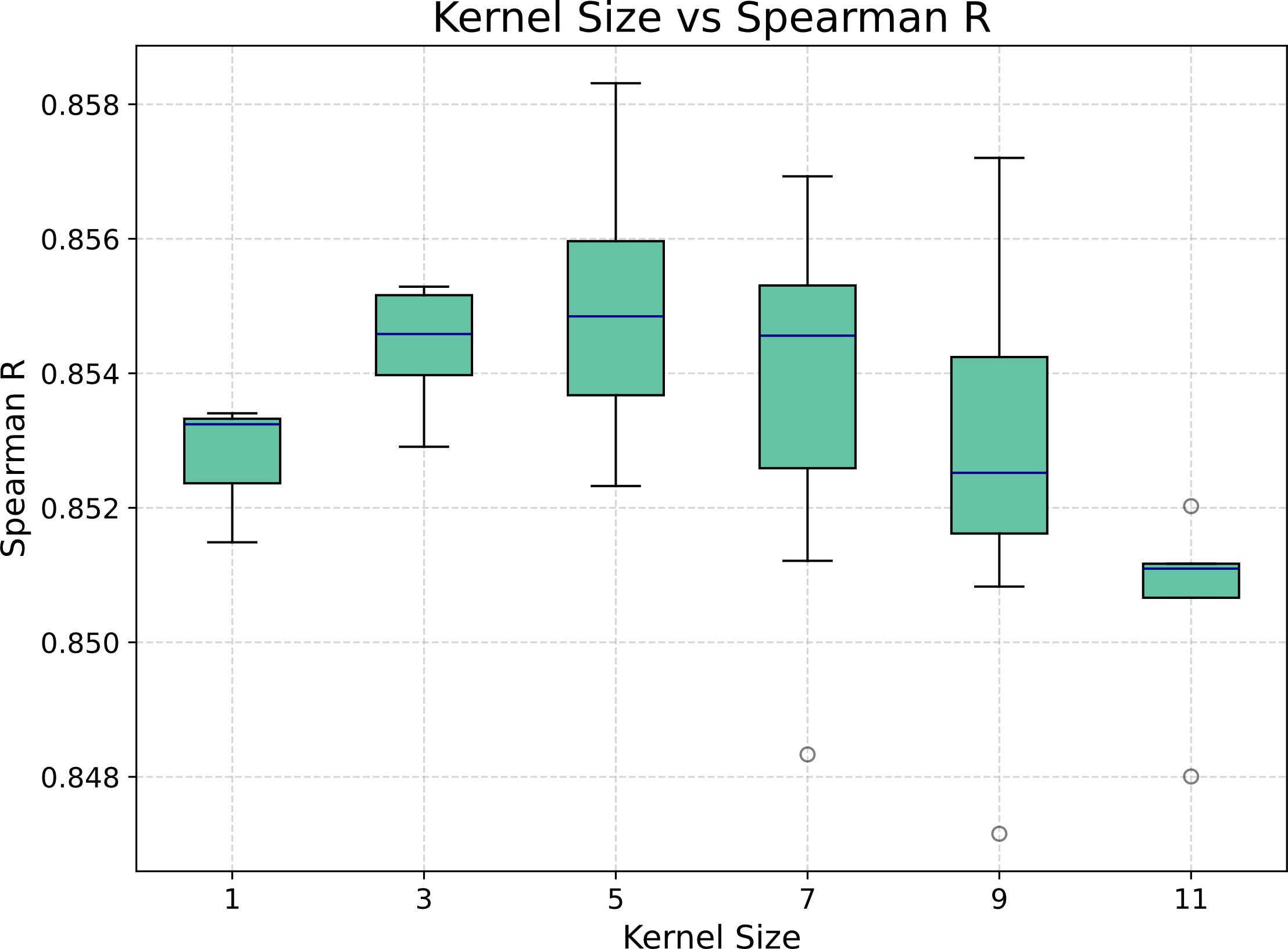


**Fig. S3** Ablation study of convolution kernel size (*k*) in the Conv-Attention model. The boxplot shows the distribution of Spearman R values across trials with different kernel sizes. The configuration with *k = 5* achieves the best trade-off between performance and stability, and is thus chosen for the final model.

### 4. Training loss curve


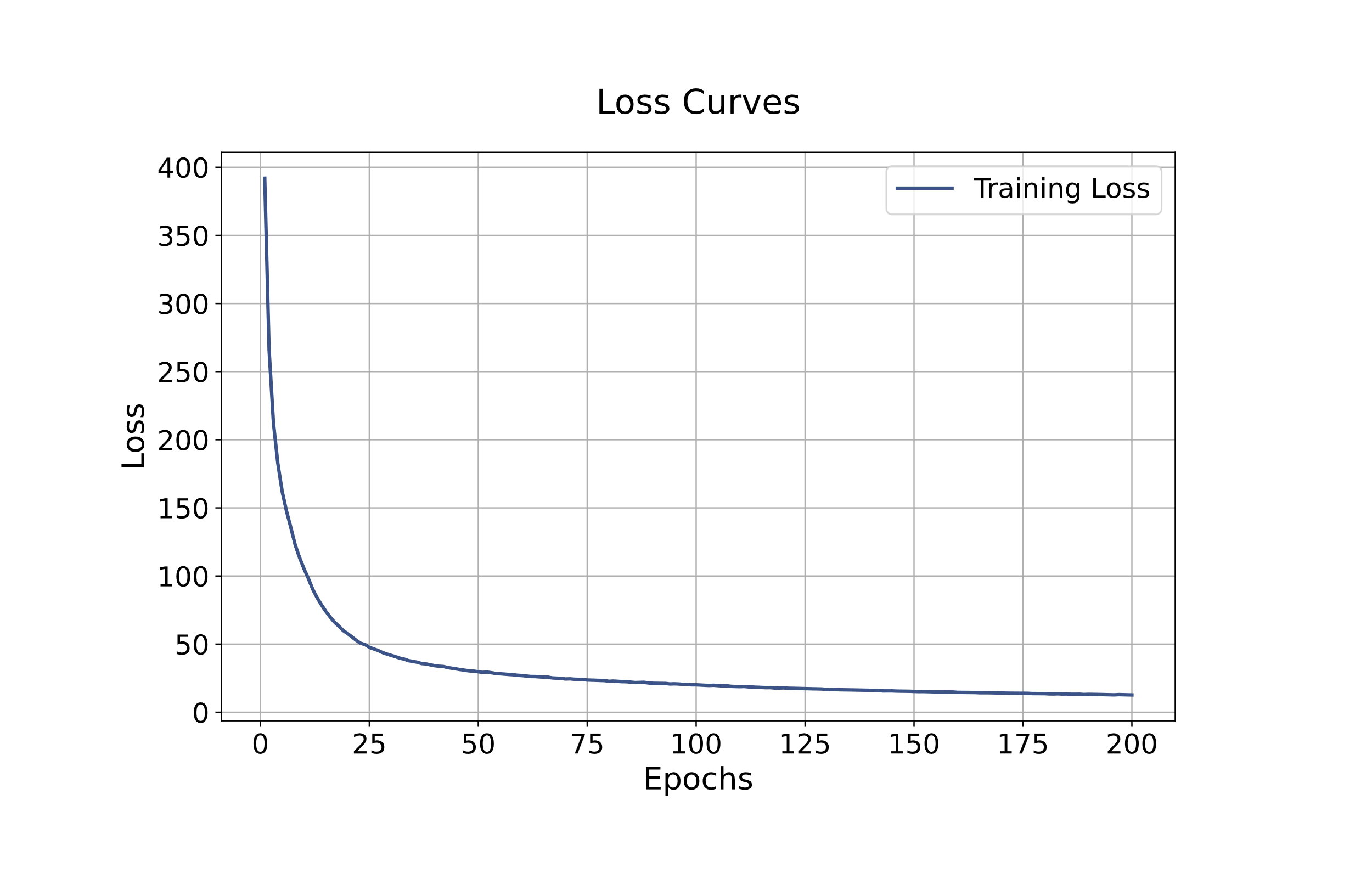


**Fig. S4** Loss curve. The plot demonstrates a consistently smooth decline in loss throughout the training process.

### 5. Optuna hyperparameters tuning

**Table S1. Hyperparameter Search Space**

| **Hyperparameter** | **Range/Type** | **Sampling Method** | **Step** |
| --- | --- | --- | --- |
| conv1_channels | 16–192 | Uniform integer | 16 |
| conv2_channels | 16–192 | Uniform integer | 16 |
| conv3_channels | 16–192 | Uniform integer | 16 |
| lookahead_k | 1–10 | Uniform integer | 1 |
| lookahead_alpha | 0.0–1.0 | Uniform float | – |
| dropout1 | 0.0–0.5 | Uniform float | – |
| dropout2 | 0.0–0.5 | Uniform float | – |
| dropout3 | 0.0–0.5 | Uniform float | – |
| learning_rate | 1e-5–1e-2 | Log-uniform float | – |
| fc1_shared_out | 128–1536 | Uniform integer | 32 |
| fc1_branch_out | 32–768 | Uniform integer | 16 |
| use_conv_attn0 | True/False | Boolean | – |
| use_conv_attn1 | True/False | Boolean | – |
| use_conv_attn2 | True/False | Boolean | – |
| use_conv_attn3 | True/False | Boolean | – |
| use_channel_attn0 | True/False | Boolean | – |
| use_channel_attn1 | True/False | Boolean | – |
| use_channel_attn2 | True/False | Boolean | – |
| use_channel_attn3 | True/False | Boolean | – |

**Table S2. Optimal Hyperparameter Configuration Selected by Optuna**

| **Hyperparameter** | **Optimal Value** |
| --- | --- |
| conv1_channels | 112 |
| conv2_channels | 144 |
| conv3_channels | 144 |
| lookahead_k | 5 |
| lookahead_alpha | 0.66 |
| dropout1 | 0.3 |
| dropout2 | 0.002 |
| dropout3 | 0.2 |
| learning_rate | 8 × 10⁻⁴ |
| fc1_shared_out | 800 |
| fc1_branch_out | 192 |
| use_conv_attn0 | True |
| use_conv_attn1 | True |
| use_conv_attn2 | True |
| use_conv_attn3 | True |
| use_channel_attn0 | False |
| use_channel_attn1 | False |
| use_channel_attn2 | False |
| use_channel_attn3 | True |

**6. Protospacer sequence features optimization**

Building upon the gradient ascent framework, we applied the same method to the protospacer region (nucleotide positions 10–29) of prime editing, focusing on the four base input channels (A/G/T/C) to reveal the ideal protospacer sequence under maximization of the target outputs. During optimization, the activation value of each base at each position represents its relative probability under each output condition (Validly Edited, Unedited, Erroneously Edited). Finally, we used Logomaker to construct SeqLogos based on these activation probabilities, visually displaying the optimal base composition of the protospacer region for each of the three target outputs.


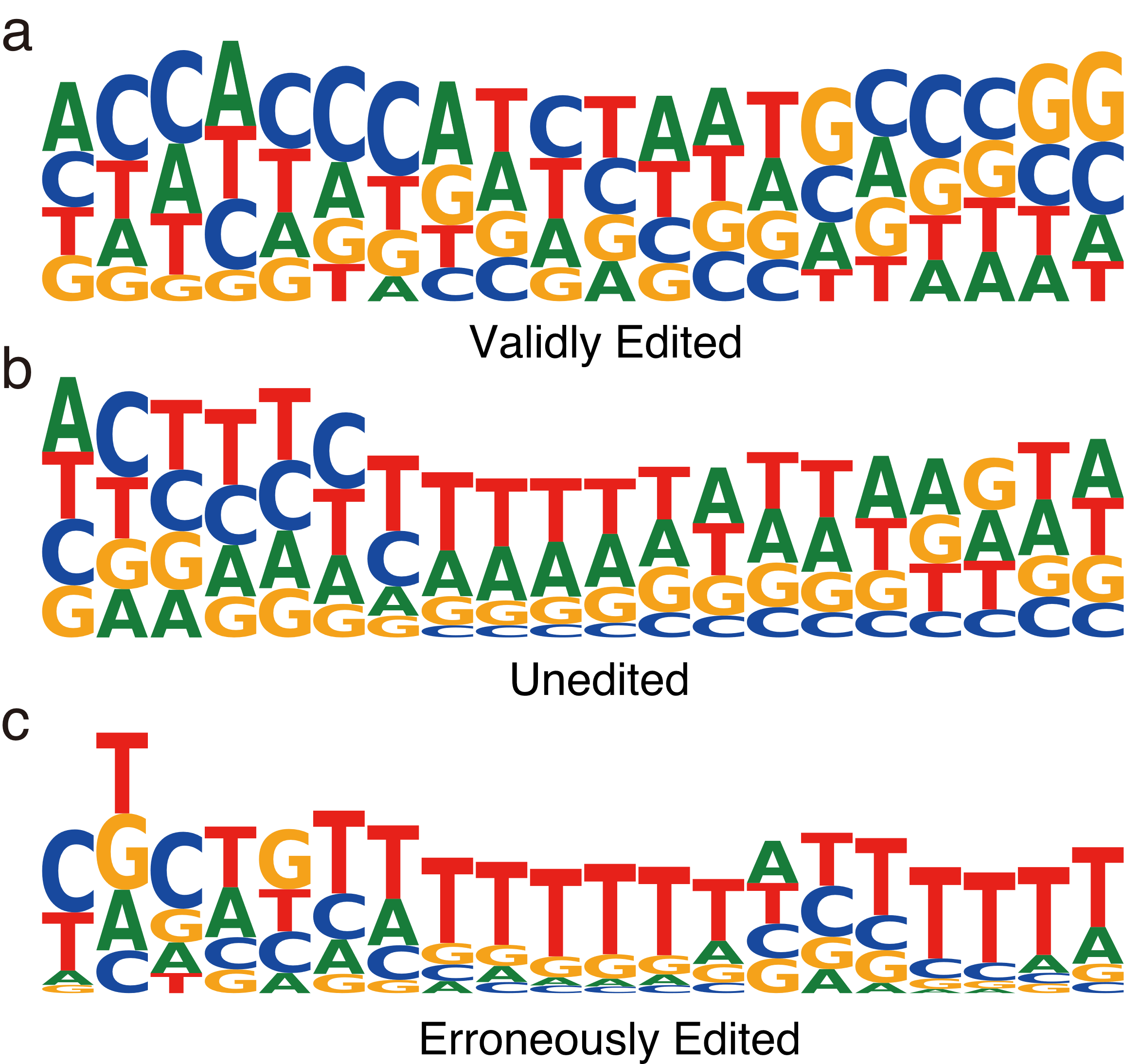


**Fig. S5** Sequence Logo of protospacer sequence features. Letter heights reflect the relative activation probabilities of each base at each position under maximization of the respective target output, depicting the optimal protospacer sequence design. a Validly Edited. b Unedited. c Erroneously Edited.

**7. Ablation study of features and architecture**

We conducted ablation experiments on the K562 dataset to systematically evaluate the contributions of various components of the PrimeNet model to its predictive performance on editing efficiency. **Figure S5** presents the Spearman correlation coefficients for different model variants, each corresponding to the removal or modification of a specific input feature or architectural module.

The K562 dataset was chosen for this ablation analysis based on initial observations regarding the difference in dataset sizes between the two cell lines. The HEK293T dataset contains 107,165 samples, while K562 contains only 18,742 samples—a ratio of approximately 5.7:1. In our preliminary experiments, we observed that the model maintained strong performance on HEK293T even when certain regulatory features were removed. This is likely due to the larger dataset size enabling the model to adapt effectively using sequence information alone. In contrast, the more limited sample size of K562 makes the model more dependent on cell-type-specific regulatory features such as epigenetic data. This trend is already reflected in **Figures 3 and 4** of the main text, which motivated our decision to conduct the full ablation study on the K562 dataset, where the influence of such features can be more clearly observed.

The results show that removing epigenetic features (*no_epigenetic*) significantly impairs the model’s performance, highlighting their essential role in modeling under low-data conditions. We also evaluated two architectural modules—multi-scale representation (*no_multi_scale*) and multi-branch structure (*no_multi_branch*). While removing these components also led to performance degradation, the impact was less severe compared to the exclusion of epigenetic features. These findings suggest that, for this task, the diversity of data and inclusion of biologically relevant annotations have a greater influence on model performance than architectural refinements alone.


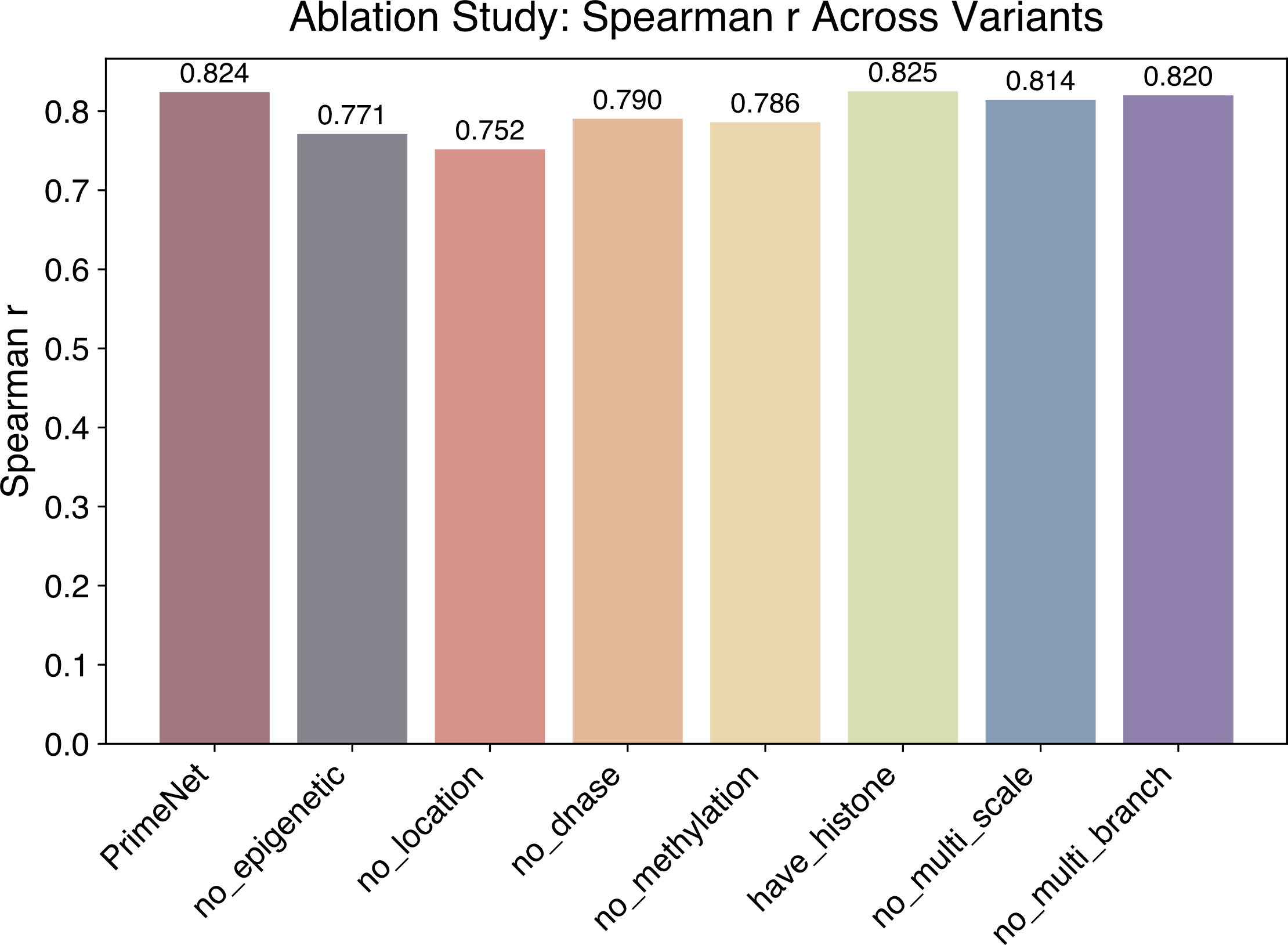


**Fig. S6 Ablation study of PrimeNet on Spearman correlation of valid edits.** The bar plot presents the Spearman correlation coefficients between predicted and observed editing efficiencies for valid edits across different ablation variants of PrimeNet. The baseline model (PrimeNet) is compared to versions with specific components removed, including epigenetic features (no_epigenetic), positional encoding (no_location), DNase signals (no_dnase), methylation (no_methylation), multi-scale modules (no_multi_scale), and multi-branch structures (no_multi_branch). Additionally, a version with only histone features retained (have_histone) is included. The results highlight the contribution of each component to the overall predictive performance.

**8. Performance of models based on embeddings from** **large pretrained DNA models**

**Table S3. Performance of Generator and Evo2 embeddings with XGBoost on prime editing prediction**

| **Model** | **Cell Line** | **Outcome** | **Spearman’s R** | **Pearson’s r** |
| --- | --- | --- | --- | --- |
| **Generator** | Hek293T | Validly Edited | 0.5082 | 0.5349 |
|  |  | Unedited | 0.5608 | 0.5988 |
|  |  | Erroneously Edited | 0.5273 | 0.4720 |
| **Generator** | K562 | Validly Edited | 0.6572 | 0.4806 |
|  |  | Unedited | 0.5860 | 0.4965 |
|  |  | Erroneously Edited | 0.2189 | 0.2867 |
| **Evo2** | Hek293T | Validly Edited | 0.5542 | 0.5771 |
|  |  | Unedited | 0.6120 | 0.6430 |
|  |  | Erroneously Edited | 0.5941 | 0.5541 |
| **Evo2** | K562 | Validly Edited | 0.6313 | 0.4607 |
|  |  | Unedited | 0.5670 | 0.4872 |
|  |  | Erroneously Edited | 0.1839 | 0.2420 |
